# 

**DOI:** 10.1192/bjb.2022.67

**Published:** 2023-08

**Authors:** Barnabas J. Gilbert

**Affiliations:** Academic Clinical Fellow in Brain Sciences at Imperial College London, UK. Email: bgilbert@ic.ac.uk

**Figure:**
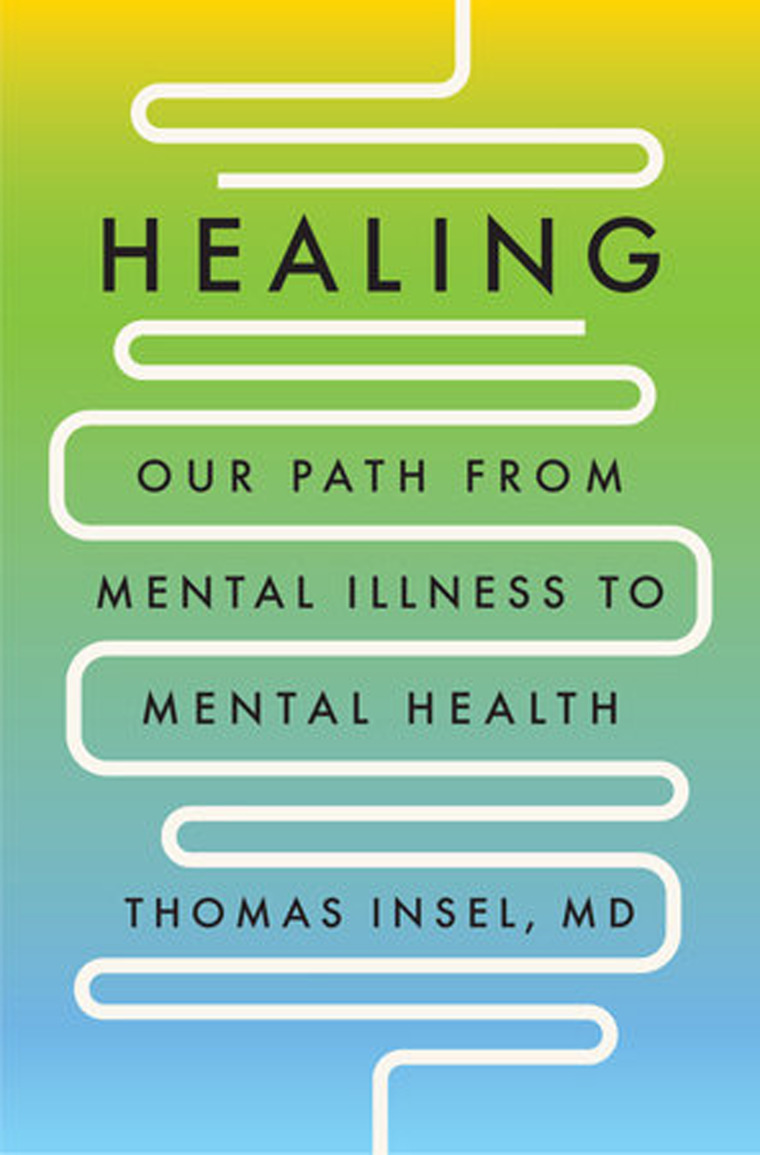

Insel shows, in this book, that he understands where clinical practice, neuroscience and emerging technologies come together. The book serves both to lament mental healthcare in America and to instil hope for its future. Insel weaves a rare narrative, built on but transcending evidence, as he shows where our current efforts to care fall short and how, in their place, an equitable system can be built.

In part, this book serves as a memoir of Insel's circuitous career, from his days as a fly-fishing psychiatry trainee in California to his work unpacking the neurobiology of social connection as a neuroscientist in the 1980s. His ability to translate basic science into real-world practice led to his appointment as Director of the National Institute of Mental Health from 2002 to 2015, a tenure coinciding with the emergence of both genomics and the internet. After channelling approximately $20 billion in public funding into the former without seeing meaningful progress, Insel was struck by the challenge that the ‘house is on fire’ yet we were thinking ‘about the chemistry of the paint’. A pragmatist at heart, Insel believes that today's most effective mental health interventions should focus on deepening human connection. This is what drove him towards the digital revolution, first leading Google's mental health function and latterly co-founding Mindstrong and Humanest Care.

Amid the louder voices of providers, insurers and pharma companies, Insel focuses on the struggling person and their family. This is easy to say but hard to do, especially in mental illness, which so often affects a person's sense of who they are: while I may *have* cancer, I *am* depressed. Insel's vision could be further developed through a sharper focus on prevention over recovery, and by laying out practical blueprints for applying this vision to our varied roles as psychiatrists, psychologists, nurses, researchers and technologists.

Ultimately, this book feels like a passing of the baton to the next generation of mental health advocates and professionals. Its lessons resound beyond America, for ‘there are only two kinds of families […]  those who are struggling with mental illness and those who are not struggling with a mental illness *yet*’. Each of us must seek out the part of the system where we can serve best, with passion and patience, if we are to bend the arc towards mental health justice.

